# Moura PL, Nannya Y, Aliouat A, et al. Competition of dual *SF3B1*^mt^ clones in MDS-RS is associated with distinct RNA mis-splicing in hematopoietic stem cells. *Blood Neoplasia.* 2024;1(2):100011.

**DOI:** 10.1016/j.bneo.2025.100088

**Published:** 2025-05-09

On page 1, the byline omits an author, Sean Wen. The byline and author affiliations should read:

Pedro Luis Moura,^1^,∗ Yasuhito Nannya,^2-4^,∗ Affaf Aliouat,^5^,∗ Isabel Juliana Hofman,^1^,∗ Teresa Mortera-Blanco,^1^ Tetsuichi Yoshizato,^1^ Ryunosuke Saiki,^2,3^ Masahiro M. Nakagawa,^2,3^ Maria Creignou,^1,6^ Sean Wen,^5^ Ann-Charlotte Björklund,^1^ Gunilla Walldin,^1^ Indira Barbosa,^1^ Monika Jansson,^1^ Francesca Grasso,^1^ Edda M. Elvarsdottir,^1^ Petter S. Woll,^1^ Sten Eirik W. Jacobsen,^1,5-7^ Seishi Ogawa,^1-3^ and Eva Hellström-Lindberg^1,6^

^1^Center for Hematology and Regenerative Medicine, Department of Medicine Huddinge, Karolinska Institutet, Huddinge, Sweden; ^2^Department of Pathology and Tumor Biology, Graduate School of Medicine and ^3^WPI Institute for the Advanced Study of Human Biology, Kyoto University, Kyoto, Japan; ^4^Institute of Medical Science, University of Tokyo, Tokyo, Japan; ^5^Hematopoietic Stem Cell Laboratory, Molecular Haematology Unit, Medical Research Council Weatherall Institute of Molecular Medicine, Radcliffe Department of Medicine, University of Oxford, Oxford, United Kingdom; ^6^Division of Hematology, Department of Medicine, Karolinska University Hospital, Huddinge, Sweden; and ^7^Department of Cell and Molecular Biology, Karolinska Institutet, Stockholm, Sweden

∗P.L.M., Y.N., A.A., and I.J.H. are joint first authors, and contributed equally to this study.

The authorship contribution statement on page 5 should include the information that Sean Wen conducted investigation and was responsible for software.

Also on page 5, the conflict-of-interest disclosure statement should mention that coauthor Isabel Juliana Hofman confirms that the research presented in this study was conducted before she joined her current employer, Elsevier, the publisher of *Blood Neoplasia*. Additionally, the peer review process was carried out independently of her affiliation.

